# Ion-channel aligned gas-blocking membrane for lithium-air batteries

**DOI:** 10.1038/s41598-017-12207-8

**Published:** 2017-09-20

**Authors:** Wonsung Choi, Mokwon Kim, Jung Ock Park, Joon-Hee Kim, Kyunghwan Choi, Yong Su Kim, Tae Young Kim, Ken Ogata, Dongmin Im, Seok-Gwang Doo, Yunil Hwang

**Affiliations:** 1 Samsung Advanced Institute of Technology, Samsung Electronics, Suwon, 16678 Republic of Korea; 2 Samsung Research Institute of Japan (SRJ), Samsung Electronics, Osaka, 562-0036 Japan

## Abstract

Lithium-metal-based batteries, owing to the extremely high specific energy, have been attracting intense interests as post-Li-ion batteries. However, their main drawback is that consumption/de-activation of lithium metal can be accelerated when O_2_ or S used in the cathode crosses over to the metal, reducing the lifetime of the batteries. In use of ceramic solid state electrolyte (SSE) separator, despite the capability of gas blocking, thick and heavy plates (~0.3 mm) are necessitated to compensate its mechanical fragility, which ruin the high specific energy of the batteries. Here, we demonstrate fabrication of a new membrane made of micron-sized SSE particles as Li-ion channels embedded in polymer matrix, which enable both high Li-ion conduction and gas-impermeability. Bimodal surface-modification was used to control the energy of the particle/polymer interface, which consequently allowed channel formation via a simple one-step solution process. The practical cell with the new membrane provides a cell-specific energy of over 500 Wh kg^−1^, which is the highest values ever reported.

## Introduction

**Figure 1:**
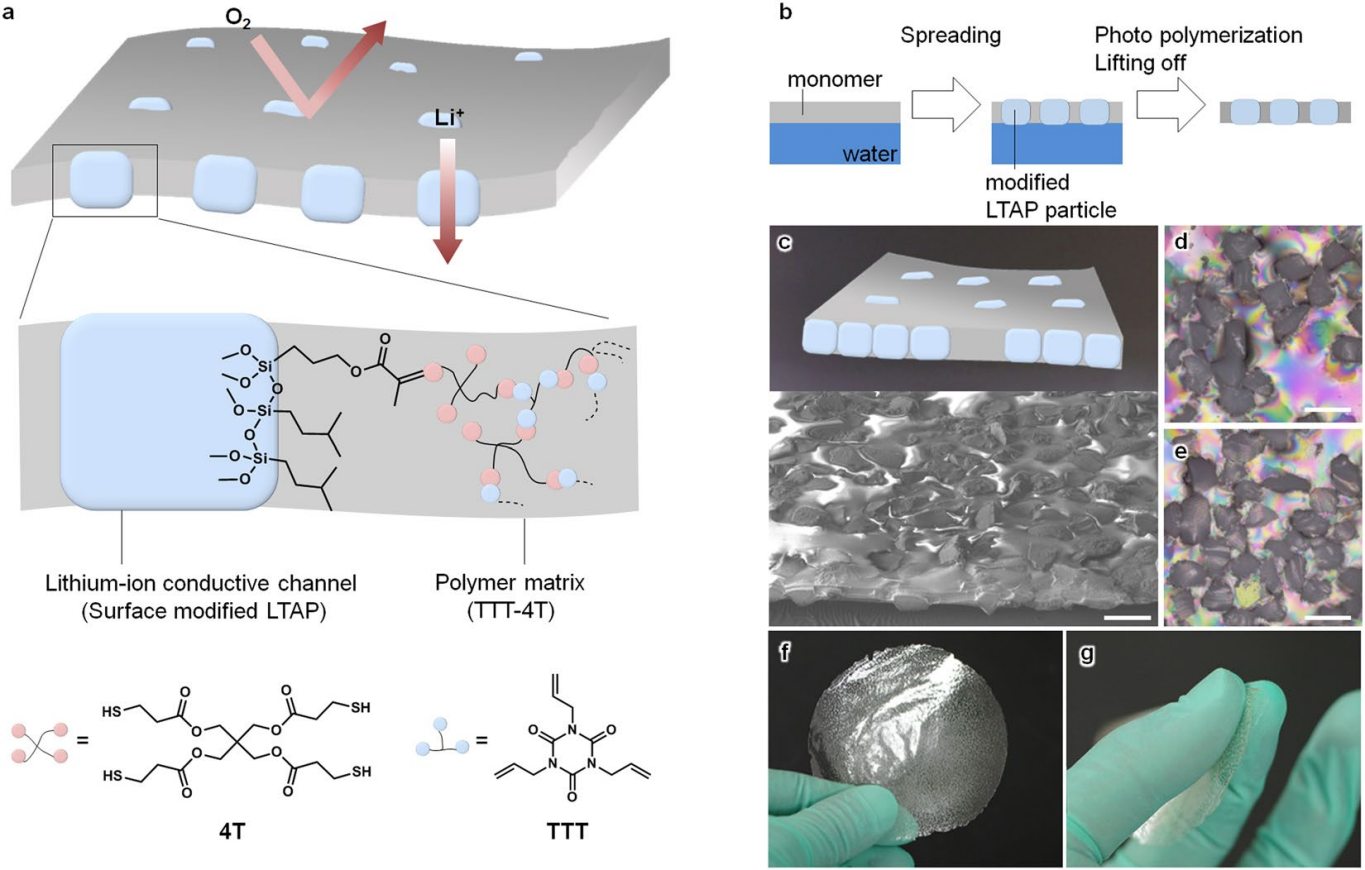
(**a**) Schematic illustration of the membrane with aligned lithium-ion conducting channel. (**b**) Schematic illustration of the composite membrane preparation. (**c**) A perspective SEM image of the composite membrane. (**d**), (**e**) Polarising optical micrographs of the IB-PM-LATP composite membrane: (**d**) top view and (**e**) bottom view. (**f**), (**g**) Photographs showing the composite membrane (8 cm in diameter).

High theoretical specific capacity of lithium (3861 mAh g^−1^) has inspired researchers to push the limit of Li-ion batteries by developing lithium-metal-based batteries^1^, which are even more attractive when combined with oxygen or sulfur cathodes^2,3^. However, in these cases, the crossover of O_2_/S to the to the metal side, dendrite growth, and unnecessary electrolyte decomposition should be addressed for battery safety and durability^4–7^. In Li-air batteries, a monolithic Li-ion conducting ceramic could be inserted as a gas blocking separator to protect the metal from corrosion^8–25^. This ceramic membrane also completely separates the anode and cathode sides, allowing the use of different electrolyte compositions on each side^6^. Carefully selected electrolytes can suppress excessive reactions on the metal, and this allows the uses of mediators^8–10^ and aqueous electrolytes on the cathode^11–23^. However, thick ceramic plates are needed to compensate for their mechanical fragility, which adds significant volume and mass to the cell and lowers the specific energy of the Li-air batteries. For example, the typical areal weight of the ceramic membrane (88 mg cm^−2^ for 260 μm thickness) is much greater than that of the cathodes (0.5–5 mg cm^−2^)^1,21^. Even assuming infinite conductance for the 260-μm-thick ceramic membrane and the use of 100 μm-thick cathode, the cell output may not exceed 500 Wh kg^−1^ (see the Supplementary Information Fig. S1 for detailed cell properties, which are estimated based on ref.^21^). In contrast, much thinner ceramic membranes^14^ face difficulty in scaling-up due to their fragile nature. Instead of the ceramic membranes solid polymer electrolytes, such as polyethylene oxide-based polymers has also been suggested as the protecting separator, yet it could react with the cathode material and have poor gas/electrolyte-barrier properties^26–30^. Here, we present a new design of Li-ion conducting membrane in which micron-sized solid-state electrolyte (SSE) particles as Li-ion conducting channels are self-aligned in a polymer matrix via a simple one-step solution process (Fig. 1). A thiol-ene network polymer is chosen as the polymer matrix to provide an adhesive surface for surface-modified ceramic particles, leading to low gas permeability. An area-specific resistance value of 24 Ω cm^2^ is achieved for the composite membrane at 60 °C by decreasing the thickness and increasing the packing density of the ceramic particles in the membrane. The areal weight of the thinnest composite membrane (2.4 mg cm^−2^), represents a 30-fold reduction compared to that of a commercially available ceramic plate membrane. We demonstrated the operation of a Li-air rechargeable battery using the composite membrane to achieve a cell-specific energy of more than 500 Wh kg^−1^.

## Fabrication of the Membrane

The composite membrane depicted schematically in Fig. 1a has Li-ion conducting channel through the SSE particles that are exposed on both top and bottom sides of the polymer matrix. Previously, we and other groups have developed Li-ion conducting channel by removing the polymer coated on the channel via destructive mechanical polishing^31^ and chemical etching processes^32^. In contrast, the preparation method presented here (Fig. 1b), used a modified float-casting method^33^, providing good adhesion between the SSE particles and the polymer matrix which is essential to avoid the undesirable crossover of materials between the cathode and the anode sides. In mixed matrix membranes, interface between polymer and additive often the determinant of the barrier properties, as the polymer detachment from the additive creates voids that increase the permeability^34,35^. A low-shrinkage, photo-curable polymer composed of a multifunctional thiol (pentaerythritol tetrakis(3-mercaptopropionate), 4T), and a multifunctional ene (1,3,5-triallyl-1,3,5-triazinane-2,4,6-trione, TTT) was chosen as the polymer matrix (Fig. 1a) to avoid the formation of these voids^36,37^. The thiol-ene polymer can react with the surface-modified particles to form a highly cross-linked network, leading to high gas barrier properties. A solution of the monomer was cast onto a water surface, and the solvent was evaporated to form a monomer layer. Lithium aluminium titanium phosphate-based glass-ceramic (LATP, Li_1+x+y_Al_x_(Ti,Ge)_2−x_Si_y_P_3−y_O_12_) particles were modified with silane compounds and sprinkled onto the monomer layer using a standard sieve as SSE particles. Careful tuning of the surface-modification allowed the LATP particles to spontaneously form open channels in the monomer layer. The composite layer was then photo-cross-linked and lifted from the water surface to yield a free-standing composite membrane, as shown in Fig. 1c–e. There was no polymer coverage on the top and bottom side of the LATP particles, avoiding the destructive processes to remove the over-coated polymer^31,32,38^. The size of the membrane was mainly determined by the size of the water bath, and the composite membrane, shown in Fig. 1f and g, was 8 cm in diameter. The composite membrane was also sufficiently flexible to improve the freedom of design^39–41^.

LATP was used as the main component of the Li-ion conducting channel because of its chemical stability and its high Li-ion conductivity, as reported elsewhere^8–11,13–18,20–25^. A plate of LATP was pulverised and sieved into uniformly sized particles. Then, the LATP particles were modified with 3-(trimethoxysilyl)propyl methacrylate (PM) and isobutyl(trimethoxy)silane (IB), yielding PM-LATP and IB-LATP, respectively. The PM-LATP particles were further modified with IB to yield IB-PM-LATP particles. The surface modification of LATP particles was confirmed by X-ray photoelectron spectroscopy (XPS, Fig. S4). Surface and cross-sectional images of the composite membranes prepared from TTT-4T and IB-PM-LATP are illustrated in Fig. 1b–f, showing the channel structure of the LATP penetrating the polymer matrix.

**Figure 2:**
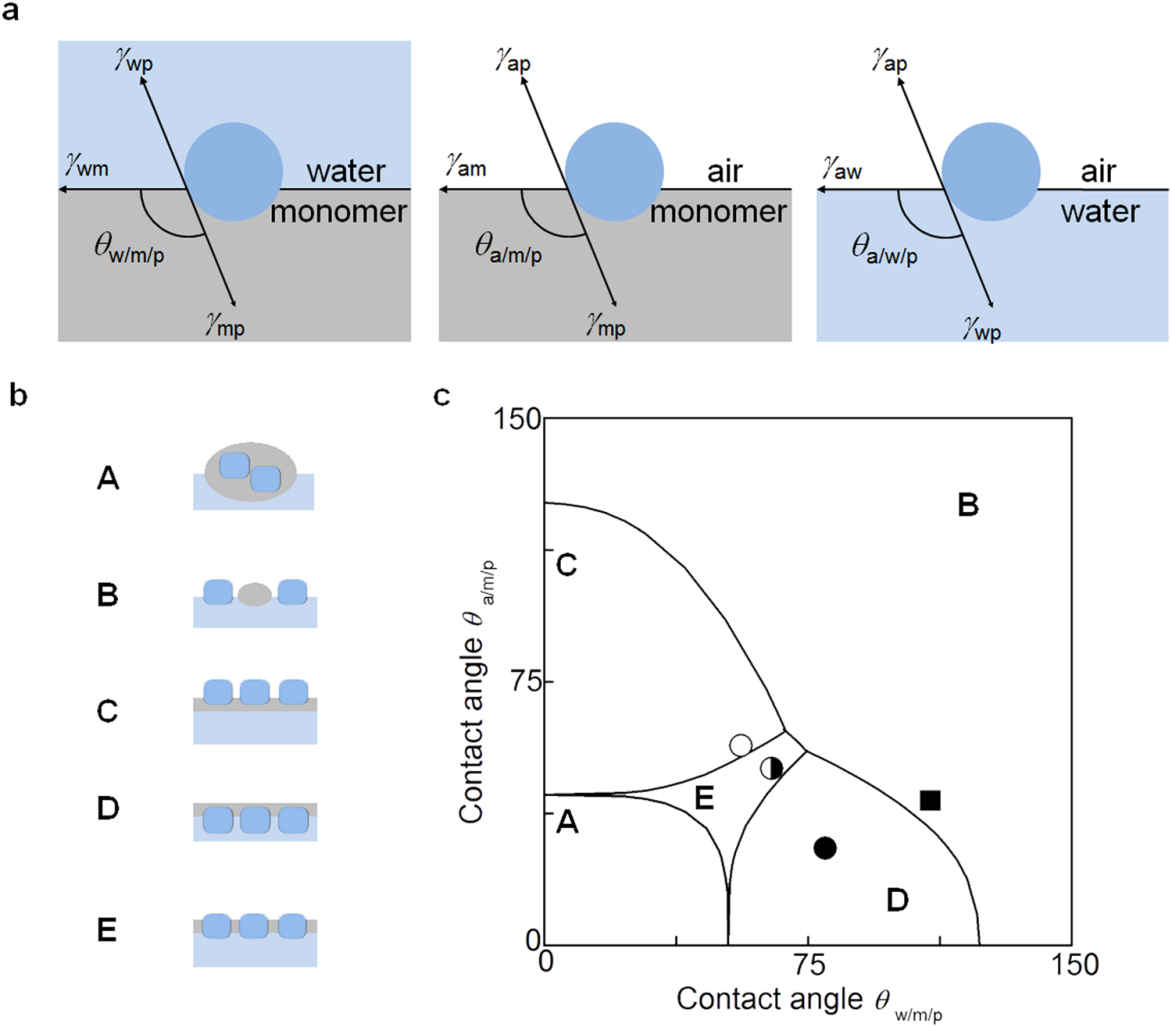
(**a**) Contact angles of the particle at the water/monomer (*θ*_w/m/p_), air/monomer (*θ*_a/m/p_), and air/water (*θ*_a/w/p_) interfaces. (**b**) Possible structural models for the float-casting method. (**c**) Phase diagram of the possible structural models showing results for IB-LATP (empty circle), PM-LATP (filled circle), IB-PM-LATP (half-filled circle), and LATP (square).

We adopted a phase diagram^42,43^ (Fig. 2a) using the contact angles of the monomer on the LATP particles in water (*θ*_w/m/p_) and that in the air (*θ*_a/m/p_), in order to elucidate the effect of the surface modification of LATP particles on the membrane structure and provide a predictive tool. When the monomer layer was thinner than the LATP particles, the membrane structure can be divided into five structural models: A, B, C, D, and E as shown in Fig. 2b, E representing the channel structure. The phase diagram of the energetically favourable membrane structures was derived from surface energies calculated from the measured contact angles (see Supplementary Information for further details). The phase boundaries were drawn using coexistence conditions based on the ratio between interfacial tensions among the monomer, water, and air (*γ*_am_/*γ*_aw_ = 0.69 and *γ*_wm_/*γ*_aw_ = 0.33) to give five phases that correspond to the five structural models in Fig. 2c.

**Figure 3:**
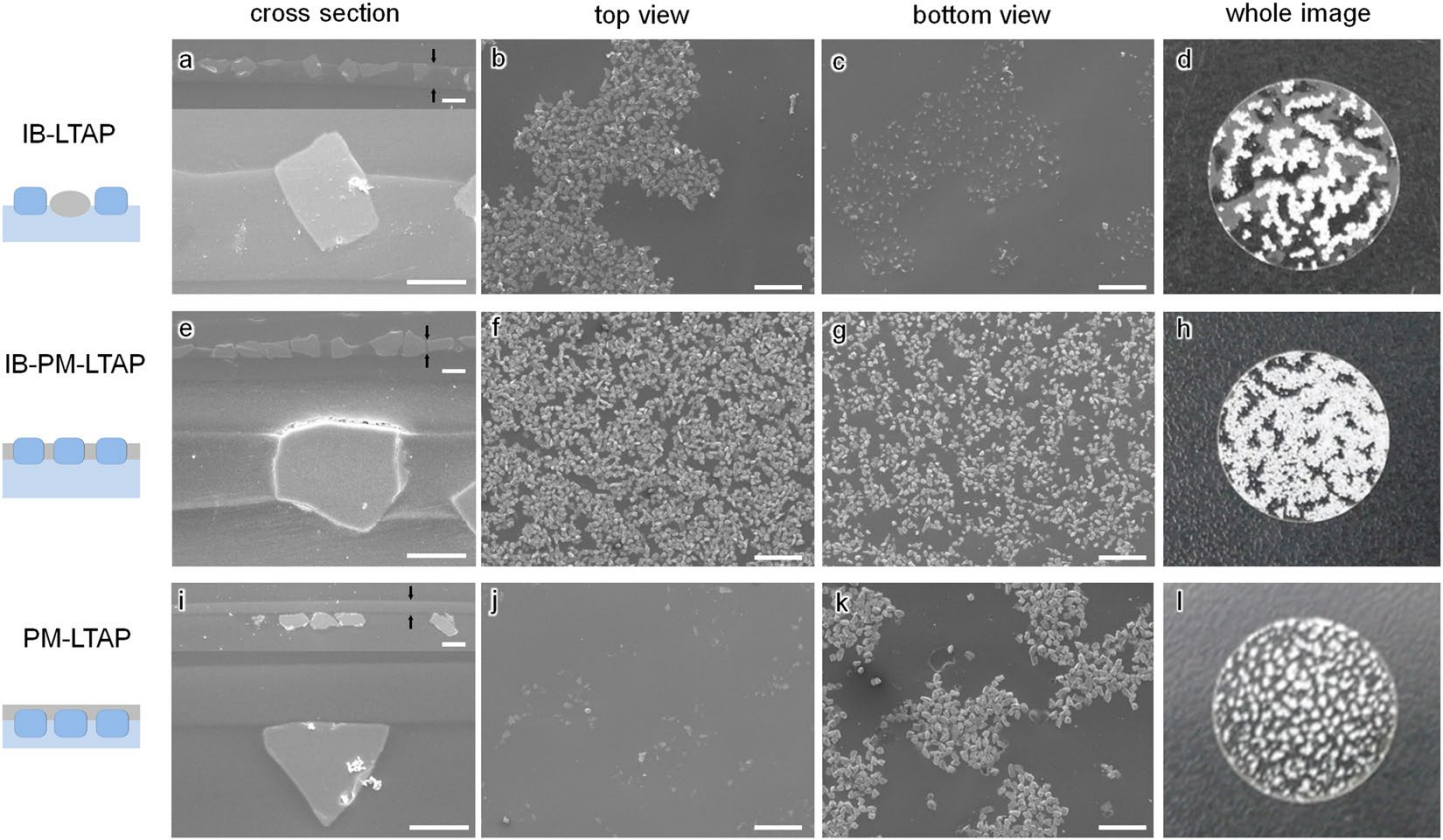
SEM images of composite membranes containing IB-LATP (**a**–**d**), IB-PM-LATP (**e**–**h**), and PM-LATP (**i**–**l**). Scale bar: (**b**,**c**,**f**,**g**,**j**,**k**) 500 μm, and (**a**,**e**,**i**) 25 μm.

The experimentally obtained contact angles *θ*_a/m/p_ and *θ*_w/m/p_ for the surface-modified and unmodified LATP plates are plotted in the phase diagram in Fig. 2c. Correspondingly, the prepared LATP, PM-LATP, IB-LATP, and IB-PM-LATP particles were dispersed in the monomer layer and cured with a UV cross-linker. A 16 mm diameter disk of the resulting cross-linked composite membrane was punched out and imaged using scanning electron microscopy (SEM, Fig. 3). Cross-sectional images were obtained by mounting a composite membrane sample in an epoxy resin. Since the *θ*_w/m/p_ and *θ*_a/m/p_ values of the unmodified LATP plate (110° and 41°, respectively) indicate that the composite membrane using unmodified LATP has structure type B in the phase diagram of Fig. 2c. When unmodified LATP particles were dispersed in the monomer layer, the particles sunk in water to maximise the interactions between the highly hydrophilic metal oxide surface and water. This separation of the particles from the monomer layer resulted in the formation of a membrane with structure B. The PM-LATP and IB-LATP particles exhibited structure types D (black circle in Fig. 2c, *θ*_w/m/p_ = 80°, *θ*_a/m/p_ = 28°) and C (empty circle in Fig. 2c, *θ*_w/m/p_ = 56°, *θ*_a/m/p_ = 57°), respectively. These data indicate that the PM coating enhanced the hydrophilicity of LATP, while the IB coating made LATP more hydrophobic. Thus, the PM-LATP particles tended to adsorb at the monomer/water interface, while the IB-LATP particles tended to adsorb at the air/monomer interface.

**Figure 4:**
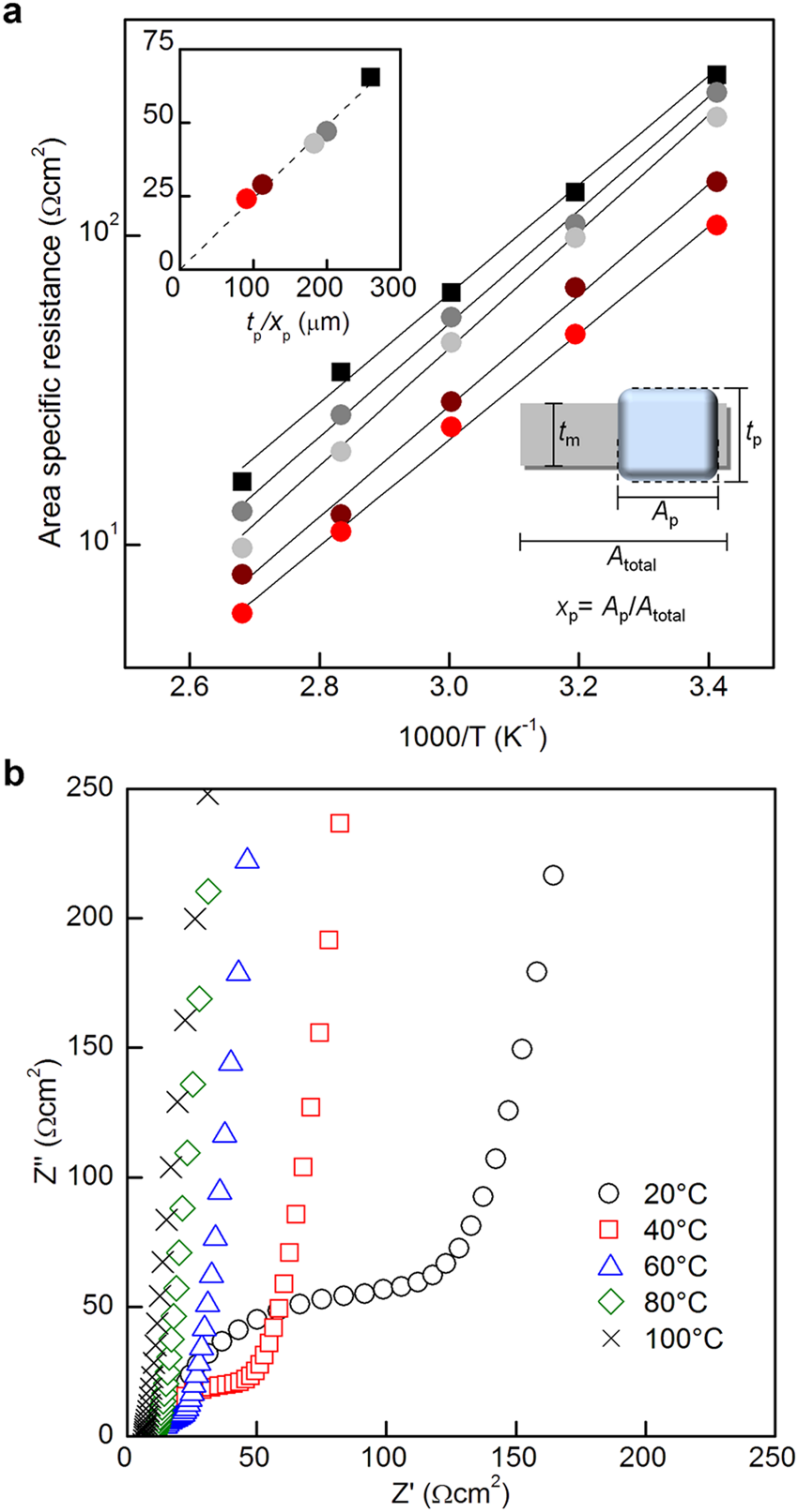
(**a**) Area-specific resistance plots of the IB-PM-LATP composite membrane. (inset) Area-specific resistance plots as a function of *t*_p_/*x*_p_. The thickness and partial area of the IB-PM-LATP particles (*t*_p_, *x*_p_) are, respectively: 260 μm, 1.0 (black); 69 μm, 0.30 (dark grey); 64 μm, 0.32 (grey); 45 μm, 0.36 (wine red); and 20 μm, 0.23 (red). (**b**) Nyquist plots of the 20-μm composite membrane.

When PM-LATP particles were spread on the monomer layer, the particles and the monomer spontaneously reorganised and were then photo-crosslinked to preserve the particles’ aligned configuration. SEM images of the PM-LATP composite membrane are shown in Fig. 3i–l. The cross-sectional image in Fig. 3i shows that the PM-LATP particles were laterally aligned and located beneath the polymer layer, indicating that these particles had adsorbed at the monomer/water interface, which is inconsistent with structure D in Fig. 2c. The bottom view of the PM-LATP composite membrane in Fig. 3k shows that the particles aggregated in small clusters that further formed larger clusters, and that no particles were observed in the top view. The composite membranes containing more hydrophobic IB-LATP were prepared in the same way as the PM-LATP membranes, and their cross-sectional images in Fig. 3a show that it had structure type C, with the IB-LATP particles located above the polymer layer. These images indicate that the IB-LATP particles adsorbed at the air/monomer interface. The top view of the IB-LATP-containing membrane in Fig. 3d further shows that the particles aggregated into worm-like clusters with large spaces in between. The particle occupancy, *x*_p_, defined as the ratio of the total LATP particle area, *A*_p_, over the area of the composite membrane, *A*_total_, was measured using the SEM images (Fig. 4, inset) with *x*_p_ < 0.20 for the IB-LATP membrane and *x*_p_ = 0.31 for the PM-LATP membrane (Fig. 3k). While neither the PM- nor IB-modified LATPs gave contact angles that are consistent with the E phase, we found that sequentially co-modified IB-PM-LATP plates gave the desired E phase structure (half-filled circle in Fig. 2c, *θ*_w/m/p_ = 65*°* and *θ*_a/m/p_ = 50*°*). The cross-sectional SEM image of a membrane containing IB-PM-LATP particles, shown in Fig. 3e, confirmed that the particles spanned the polymer layer and were adsorbed at both the air/monomer and monomer/water interfaces. The membrane structures prepared with surface-modified LATP particles agreed well with the predictions from the surface energies. Moreover, the top view in Fig. 3f shows that the IB-PM-LATP particles were homogeneously and densely aligned with *x*_p_ = 0.36, a value that is much higher than those of composite membranes with PM-LATP or IB-LATP.

## Li-ion Conductivity

To test the area-specific resistance of IB-PM-LATP-containing composite membranes, membranes with average thicknesses of 260, 69, 64, 45, and 20 μm were sandwiched by gold electrode applied by sputter coater to assemble symmetric electrodes. The Nyquist plots of the cell of the 20-μm composite membrane in the rage from 20 °C to 100 °C are shown in Fig. 4a. The semicircle and a spike at ≤60 °C are characteristics of ionic conductors, corresponding to the bulk, grain boundary and the electrode polarisation of the solid electrolyte. When the impedance measurement was carried out for the composite membrane sandwiched between electrolytes, the Nyquist plots shows a semicircle and a spike with a characteristic frequency similar to that of the LATP plate (see Supplementary Information Fig. S5), indicating Li-ion transport through the IB-PM-LATP particles. The area-specific resistance values were calculated from the intercept of the spike, and the value of the thinnest composite membrane (particle thickness *t*_p_ = 20 µm, *x*_p_ = 0.23) is 24 Ω cm^2^ at 60 °C which is much lower than that of the LATP plate (66 Ω cm^2^, *x*_p_ = 1, *t*_p_ = 260 µm). The Li-ion conductivity of the composite membrane was calculated to be 8.3 × 10^−5^ S cm^−1^, and that of the IB-PM-LATP particles, *σ*_p_, was determined from *σ*_p_ = *t*_p_/*A*_p_ *R* to be 3.5 × 10^−4^ S cm^−1^. The latter value was in good agreement with that of the LATP plate (3.5 × 10^−4^ Scm^−1^). Figure 4a inset shows that the area specific resistances of the composite membranes, *R*, is proportional to the geometric parameters *t*_p_ and inverse proportional of *x*_p_, implying that the IB-PM-LATP particles are satisfactory exposed to the surface to conduct Li-ions without current-leaking. The areal weight of the thinnest membrane (*x*_p_ = 0.23) is 2.4 mg cm^−2^, which is remarkably low compared to that of the LATP plate. The poreless Li-ion conducting channel structure reduces both the area specific resistance and areal weight of the membrane.

## Gas Barrier Property of the Composite Membrane

**Figure 5:**
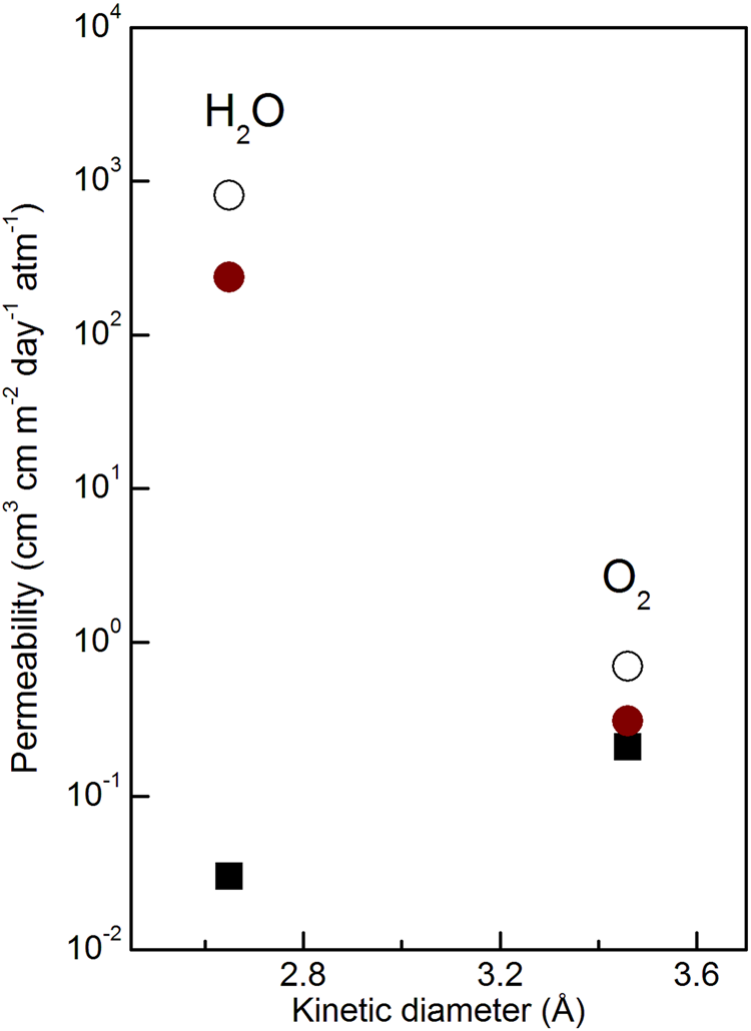
Permeability to oxygen and water vapour of the LATP plate (square), the composite membrane (red circle), and TTT-4T film (empty circle).

Since the protective separator in Li-air battery should be impermeable to gaseous cathode materials, a high gas barrier property is required for the membranes. However, the permeability of the gas has not been discussed in previous reports. The composite membrane containing IB-PM-LATP particles, the matrix polymer TTT-4T, and LATP plate were set separately on holders, and their permeability for oxygen and water vapour was determined. It should be noted that the fact that oxygen permeability measurements were successfully performed with the composite membrane corroborates the absence of pinholes, since even on pinhole on the composite membrane will cause measurement failure due to the high sensitivity of the OTR equipment over the 0.0005–200 cm^3^ m^−2^ day^−1^ test range. The measured permeability data are shown in Fig. 5. The oxygen and water vapour permeabilities are 0.70 and 810 cm^3^ cm m^−2^ day^−1^atm^−1^ for TTT-4T, and 0.21 and 0.03 cm^3^ cm m^−2^ day^−1^ atm^−1^ for the LATP plate. These high gas barrier properties are similar to the previous report^37^. The composite membrane containing IB-PM-LATP particles exhibits a gas permeability for oxygen (0.41 cm^3^ cm m^−2^ day^−1^atm^−1^) and water vapour (623 cm^3^ cm m^−2^ day^−1^atm^−1^), which is comparable or superior to those of a commercially available gas barrier film polyethylene terephthalate (PET) (0.17 and 1670 cm^3^cm m^−2^ day^−1^ atm^−1^, respectively)^28^. The permeabilities of the composite membrane, *P*_composite_ (0.55 and 567 cm^3^ cm m^−2^ day^−1^ atm^−1^ for oxygen and water vapour, respectively) agree well with the area average values given by *P*_ave,composite_ = *x*_p_ *P*_LATP_ + (1 − *x*_p_)*P*_TTT-4T_, where *P*_LATP_ denotes the permeability of the LATP plate and *P*_TTT-4T_ denote that of TTT-4T. This indicates that the particles and polymer matrix are in close contact at the molecular level without any pinhole. Such close contact between the polymer and IB-PM-LATP is supported by the strong adhesion of the IB-PM-LATP/TTT-4T interface shown in the peeling test Fig. S9.

## Li-air Battery Cell Performance

**Figure 6:**
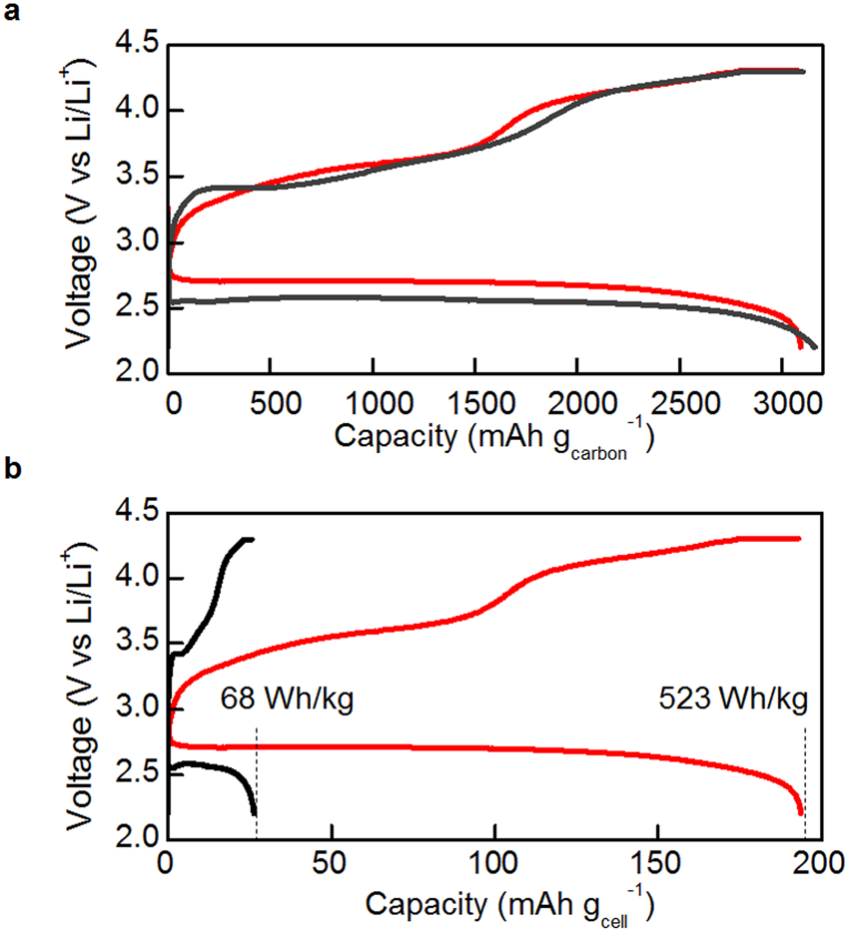
Charge-discharge curves of Li-air battery cells with the IB-PM-LATP composite membrane (*t*_p_ = 20 μm, *x*_p_ = 0.23) and the LATP plate: (**a**) capacity based on the carbon weight loaded in the cathode, and (**b**) capacity based on the cell weight.

A prototype Li-air battery with a lithium metal anode was assembled into coin cell case to evaluate the performance of our composite membrane. A mixture of multi-walled carbon nanotubes and the electrolyte was pressed into a sheet structure and punched into the cathode disc. Two different Li-air battery cells were assembled, with either the composite IB-PM-LATP membrane or the LATP plate inserted between the cathode and anode as a protecting separator. The cells were charged and discharged at 60 °C. The charge-discharge curves of the cell for the two Li-air batteries are compared in Fig. 6. Figure 6a shows charge-discharge curves for the capacity per carbon loaded in the cathode. The cell with the composite membrane exhibits a discharge plateau at 2.7 V, which is slightly higher than that of the cell with the LATP plate (2.6 V). The discharge capacity with the composite membrane achieved 3090 mAh g_carbon_^−1^, which is almost same as that of the LATP plate (3160 mAh g_carbon_^−1^). In contrast, the charge-discharge curves for the capacity based on the cell weight are shown in Fig. 6b. The specific energy density was calculated on the basis of the total weight of the cell components which include gas diffusion layer (GDL), the cathode, the catholyte layer, the protective separator, the anolyte layer, and the lithium metal anode. For the calculation, the protective separators were projected to be an 11-mm-diameter disk. According to the projection, the weight of the LATP plate and the composite membrane was estimated to be 83.6 mg and 2.3 mg, respectively. The total weight of the other components except protective separators was 10.2 mg (for a detailed summary of the cell properties see Supplementary Information Fig. S6 and Table S3). Thus the total weights of the cells with the LATP plate and the composite membrane were 94 mg and 13 mg, respectively. As shown in Fig. 6b, since the discharge capacity of the cell with the composite membrane exhibited 188 mAh g_cell_^−1^, the specific energy density of that reached 523 Wh kg^−1^, which is the highest reported value to date. This value is eight-fold greater than that of the cell with the LATP plate (68 Wh kg^−1^). The potential of the flexible protective separator to provide a much higher energy density of Li-air battery was demonstrated for the first time. The composite membrane maintained the original structure being free of defect and polymer swelling after charge-discharge cycling (the cycle performance of the cell with the composite membrane was also discussed in Fig. S7).

## Conclusion

We designed and prepared a selective Li-ion conducting membrane in which LATP particles formed Li-ion conducting channels that penetrate both side of the polymer matrix. The (in)organic hybridised membrane forms an air-tight LATP/polymer interface, in which a TTT-4T network polymer matrix is firmly bound to LATP. Bimodal surface energy modification of LATP particles with silanes enabled their spontaneous, dense alignment in the matrix, endowing gas-blocking property with lower area specific resistance (24 Ω cm^2^) and areal weight (2.4 mg cm^−2^). This overcomes the drawbacks of the current heavy and fragile ceramic membranes, allowing the maximum cell specific energy to reach over 500 Wh kg^−1^. We believe that the new design strategy, which uses a scalable solution process, will extend the commercial uses of lithium-metal-coupled rechargeable batteries. Moreover, the flexibility of the membrane can provide extra freedom in design to widen the scope of applications.

## Methods

### Materials

The photoinitiator Ignacure^®^ 369 was purchased from BASF. Multi-walled carbon nanotubes (MWCNTs) were purchased from Nanocyl SA. Polytetrafluoroethylene (PTFE) was purchased from Daikin. Lithium-ion conducting glass-ceramic plates composed of Li_2_O, Al_2_O_3_, SiO_2_, P_2_O_5_, TiO_2_, and GeO_2_ with the crystalline phase of Li_1+x+y_Al_x_(Ti,Ge)_2−x_Si_y_P_3−y_O_12_ (LATP) were purchased from Ohara Inc. (260 μm, 3.3 mg cm^−3^, and 88 mg cm^−2^). 1-Ethyl-3-methylimidazolium bis(trifluoromethylsulfonyl)imide (EMI) was purchased from Kanto Chem. Co. Pentaerythritoltetrakis(3-mercaptopropionate) (4T), 1,3,5-triallyl-1,3,5-triazine-2,4,6-trione (TTT), isobutyl(trimethoxy)silane (IB), 3-(trimethoxysilyl)propylmethacrylate (PM), tetraethyleneglycol dimethylether (TEGDME), lithium bis(trifluoromethylsulfonyl)imide (LiTFSI), and other reagents were purchased from Aldrich. All chemicals and solvents were used as received.

### Preparation of modified LATP particles

An LATP plate was pulverised and sieved using standard sieves into particles with a uniform size distribution (20–25, 38–45, 75–90, or 100–125 μm). The surface modification of the particles was carried out as follows. LATP particles (200 mg), toluene (20 mL), and IB (50 mg) were mixed and stirred for 7 h before the reaction mixture was filtered, and the solid was washed with acetone. After drying at 60 °C for 2 h, the particles were sieved again to yield IB-modified LATP particles (IB-LATP). For PM modification, LATP particles (200 mg), toluene (20 mL) and PM (50 mg) were mixed and stirred for 15 min before the reaction mixture was filtrated and washed with acetone. After drying at 60 °C for 2 h, the particles were sieved again to yield PM-modified LATP particles (PM-LATP). Finally, a mixture of IB-LATP (200 mg), toluene (20 mL), and PM (50 mg) were stirred for 30 min before the reaction mixture was filtrated and washed with acetone. After drying at 60 °C for 2 h, the particles were sieved to yield IB-PM-LATP particles.

### Membrane preparation

In a typical experiment, 4T (290 mg) and TTT (200 mg) were dissolved in a mixture (100 mg) of ethanol and chloroform (w:w = 1:1). The photo-initiator Ignacure^®^ 369 (10 mg) was added to the solution. The monomer solution (162 mg) was added dropwise to the surface of water in a Petri dish (*ϕ* = 8 cm). The thickness of the monomer layer was controlled by ensuring that the particle size was less than 90% of the lower limit size of the sieve. When particles with other sizes were used, the thickness of the monomer layer was maintained between 60–90% of the lower limit size of the sieve. The solution was slowly evaporated while half of the dish was covered with a glass cover. After evaporation, bubbles were removed from the monomer layer using a gentle flow of nitrogen. IB-PM-LATP particles (20 mg, 20–25 μm in size) were dispersed in the monomer layer before the monomer layer was irradiated with ultraviolet lamps (CL-1000, 254 nm, 10 mW cm^−2^) for 15 min to crosslink the monomer. After peeling off the cross-linked membrane from the water, a disc was punched out from the centre of the membrane for use as a composite membrane.

The areal fraction of the LATP particles in the composite membrane, *x*_p_, was estimated from the areal weight of the composite membrane, *m*_composite_, the thickness of the LATP particles, *t*_p_, the thickness of matrix polymer, *t*_m_, and their densities (*r*_p_ = 3.3 g cm^−3^, *r*_m_ = 1.30 g cm^−3^) as follows: *m*_composite_ = *x*_p_ *r*_p_ *t*_p_ + (1 − *x*_p_)*r*_m_ *t*_P_.

### Measurement of contact angles

LATP plates with dimensions of 1.5 × 1.5 cm were stirred with silane compounds in the same way as particles. The contact angles of the plates were determined using a DSA100S drop shape analyser (Krüss). The surface-modified LATP plates were dried at 60 °C before measurement. The contact angles *θ*_a/m/p_ and *θ*_w/m/p_ were determined 30 min after deposition of a mixture of TTT and 4T in air and water, respectively, and *θ*_a/w/p_ was determined 30 min after deposition of water on a surface-modified LATP plate in air. The contact angles of surface-modified LATP plates are shown in Table S2. The phase diagram classifying the structures of the composite membrane assumes that the LATP particles are spheres with uniform diameter.

### Characterisation

The surfaces of the modified LATP particles were investigated by XPS. Specimens of the composite membrane were prepared for cross-sectional imaging by placing the membranes between plastic plates and suspending the plates in epoxy, followed by curing the epoxy and polishing its surface with sandpaper and diamond powder (1 μm). Air was removed from the epoxy solution before curing. The morphology observations were performed using optical microscopy (DSX-910, Olympus) and scanning electron microscopy (SNE-4500M/MCM-100).

### Ionic conductivity

The composite membranes were masked and coated with gold by sputter coating. The AC impedance was measured between 20 and 100 °C at an amplitude of 200 mV over a frequency range from 1 Hz to 1 MHz using an impedance analyser (VMP3, Bio Logic, France). The area-specific resistance of the composite membrane was calculated from the intercept of the slanted straight line with the real axis (at temperatures above 60 °C) or, from the intercept of the spur extrapolated to the real axis (below 60 °C).

### Permeability to water vapour and oxygen

The membrane permeability to water vapour and oxygen was measured by using MOCON Aquatran model 1 and MOCON Oxytran 2/21 instruments, respectively (MOCON). The continuous-flow testing was conducted using the method described in ASTM standard D3985. Membranes with an area of 1 cm^2^ were loaded and flushed with nitrogen gas to purge excess gases and to determine a zero point. The thicknesses of the IB-PM-LATP composite membrane (*x*_p_ = 0.3), TTT-4T polymer matrix, PET, and LATP plate were 70, 78, 100, and 260 μm, respectively.

### Li-air battery cell test

A cathode was prepared by kneading a mixture of MWCNTs, PTFE, and EMI containing 0.5 M LiTFSI in a ratio of 5:1:10 by weight. The paste was then roll-pressed to prepare a cathode sheet with a loading of 2.6 mg cm^−2^. Discs (*ϕ* = 11 mm) were punched out from the prepared sheet, a carbon fibre sheet (35BA, SGL group), and lithium metal foil (30 μm) served as the cathode, GDL, and anode, respectively. A porous polymer separator (SKI, SK Innovation) was punched out into discs (*ϕ* = 11 mm) and soaked with electrolyte as the electrolyte layer: 0.5 M LiTFSI in EMI and 1 M LiTFSI in TEGDME were used as the catholyte and the anolyte. Discs (*ϕ* = 16 mm) of LATP plate (260 μm) and the IB-PM-LATP membrane (20 μm) were cut out as the protective separator. Discs of the cell components were assembled into a CR2032-type coin cell with the cell case opened at the GDL side, in the order of lithium metal anode (1.9 mg), the anolyte layer (2.0 mg), the protective separator, the catholyte layer (2.0 mg), the cathode (2.5 mg), and GDL (1.8 mg). The Li-air cell was put into a chamber, and the temperature was maintained at 60 °C. The cell was discharged at 0.05 mA cm^−2^ to 2.2 V and then charged at a current density of 0.05 mA cm^−2^ until 4.3 V. The voltage was then maintained until the current density decreased to 0.01 mA cm^−2^. The energy density was calculated on the basis of the total weight of the cell components, in which the weight of the protective separators were estimated assuming that they 11-mm-diameter disks, giving weights of 83.6 mg and 2.3 mg for the LATP plate and the IB-PM-LATP membrane, respectively. A discharge voltage of 2.7 V was used for the calculation of the specific energy density.

## Electronic supplementary material

Supplementary information
