# Supplementary material for: Ion-channel aligned gas-blocking membrane for lithium-air batteries
Source: Sci Rep. 2017 Sep 20;7:12037. doi: 10.1038/s41598-017-12207-8 (PMC5607305; doi:10.1038/s41598-017-12207-8)
Supplement: Supplementary file 1 — Supplementary information [file 41598_2017_12207_MOESM1_ESM.docx]

**Supplementary Information**

**Ion-channel aligned gas-blocking membrane for lithium-air batteries**

*Wonsung Choi, ^1^ Mokwon Kim, ^1^ Jung Ock Park, ^1^ Joon-Hee Kim, ^1^ Kyunghwan Choi, ^1^* *Yong Su Kim, ^1^* *Tae Young Kim, ^1^ Ken Ogata, ^1.2^ Dongmin Im, ^1^ Seok-Gwang Doo, ^1^ and Yunil Hwang^1^*

1. Samsung Advanced Institute of Technology, Samsung Electronics, Suwon, 443-803, Republic of Korea

2. Samsung Research Institute of Japan (SRJ), Samsung Electronics, Osaka, 562-0036, Japan

**Estimation of maximum energy density of Li-air battery**

The energy density of a Li-air cell was estimated using the data listed in Table S1 and with reference to Ref. S1. For simplicity, no gas storage tank was included. Fig. S1 shows the energy density as a function of the cathode thickness for four fixed ceramic separator thicknesses. If state-of-the-art materials are used for the cell components, such as a 100-μm cathode and a 250-μm thick ceramic separator, the energy density is calculated to be <500 Wh/kg. Reducing the thickness of the ceramic separator to 20 μm dramatically increases the energy density of a practical cell to >1100 Wh/kg, more than double that with a 250-μm thick ceramic separator.

**Fig. S1** Effect of the ceramic separator thickness on energy density of the Li-air cell as a function of cathode thickness.

**Table S1.** Cell properties used for practical cell energy calculation with reference to Ref. S1. The thickness values (*L*) correspond to the state at full charge, while the volume fraction values (*ε*) correspond to the state at the end of discharge. GDL = gas diffusion layer, CC = current collector.

**Drawing the phase diagram**

The phase diagram used to predict the structures of the composite membrane was drawn with the assumption that the LATP particles are spheres with uniform diameters. The contact angles and interfacial tensions used for this purpose are illustrated in Fig. S2.^S2^

**Fig. S2** Illustration of the contact angles and interfacial tensions.

The fluid/particle interfacial tensions (*γ*_ap_, *γ*_wp_, *γ*_mp_) can be described by the fluid/fluid interfacial tensions (*γ*_aw_,*γ*_am_, *γ*_wm_) and the contact angles (*θ*_a/w/p_, *θ*_a/m/p_, *θ*_w/m/p_) according to the Young equation:

*γ*_ap_ −* γ*_wp_ = *γ*_aw_ cos*θ*_a/w/p_ (1)

*γ*_ap_ − *γ*_mp_ = *γ*_am_ cos*θ*_a/m/p_ (2)

*γ*_wp_ − *γ*_mp_ = *γ*_wm_ cos*θ*_w/m/p_ (3)

If the particles are dispersed in a monomer layer on the water surface, they adhere to the fluid/fluid interface and form thermodynamically favourable structures A–E (Fig. S3). In that case, in each phase, the total interfacial energy obtained by adhesion to fluid/fluid interfaces could be expressed by Eq. (4)–(8),^S2^ assuming that the monomer thickness is less than the diameter of the particles.

In the phase where structure A is most favourable, the particles adhere only to the monomer.

Δ*E*_A_ = − R^2^*γ*_am_cos*θ*_a/m/p_ 

**Fig. S3** Possible structures of the composite membrane (left) and the phase diagram of the structures obtained in TTT-4T (right).

In the phase where structure B is most favourable, the particles adhere only to the air/water interface.

Δ*E*_B_ = − *R*^^*γ*_aw_(1 + cos*θ*_a/w/p_)^2^ 

In the phase where structure C is most favourable, the monomer wets the water surface, and the particles adhere to the air/monomer interface and not to the monomer/water interface.

Δ*E*_C_ = − *AS*_eq_ −*R*^^*γ*_am_(1 + cos*θ*_a/m/p_)^2^ (6)

In the phase where structure D is most favourable, the monomer wets the water surface, and particles adhere to the monomer/water interface and not to the air/monomer interface.

Δ*E*_D_ = − *AS*_eq_ −*R*^^*γ*_wm_(1 + cos*θ*_w/m/p_)^2^ − 4π*R*^^*γ*_aw_cos*θ*_a/w/p_ (7)

In the phase where structure E is most favourable, the monomer wets the water surface, and particles adhere to both the air/monomer and monomer/water interfaces.

Δ*E*_E_ = − *aAS*_eq_ − *b*{*R*^^*γ*_wm_(1 + cos*θ*_w/m/p_)^2^ – 4π*R*^^*γ*_aw_cos*θ*_a/w/p_}

− *c*π*R*^^*γ*_am_(1 + cos*θ*_a/m/p_)^2^ (8)

Here, *a* represents the monomer coverage on water. If *a* = 0.5, half the particles are adsorbed at the air/monomer interface and the other half at the monomer/water interface (E_1_). If *a* = 1, all the monomer is spread on the water surface (E_2_).

Here, *S*_eq_ and *A* represent the equilibrium spreading coefficient and the area of monomer wetting on the water surface, respectively. *AS*_eq_ is the interfacial energy obtained by wetting, and *S*_eq_ is expressed as the interfacial energy of fluid/fluid interfaces.

Δ*E* = −*AS*_eq_ = *γ*_am_ − *γ*_mw_ − *γ*_aw_ (9)

According to Eq. (1) to (3), cos*θ*_awp_ can be expressed as follows.

cos*θ*_a/w/p_ = *γ*_am_/*γ*_aw_cos*θ*_a/m/p_ − *γ*_mw_/*γ*_aw_cos*θ*_m/w/p_ (10)

Eq. (4) to (8) can be written with *θ*_a/m/p_ and *θ*_w/m/p_ from Eq. (10). Here, the contact angles (*θ*_a/w/p_, *θ*_a/m/p_, *θ*_w/m/p_) could be experimentally measured using surface-modified LATP plates (Table S2).

**Table S2.** Measuredcontact angles of surface-modified LATP plates.

Since the ratio between the two fluid/fluid interfacial tensions was determined to be *γ*_am_/*γ*_aw_ = 0.69, we have *γ*_wm_/*γ*_aw_ = 0.33 from Eq. (9) and Table S2. The phase diagram is given in Fig. S3 according to Eq. (4)–(8).

**XPS spectra of surface-modified LATP**

XPS analysis of the surface of pristine and surface-modified LATP particles was conducted, and the spectra are shown in Fig. S4a. The Si 2*p*_3/2_ spectrum of the surface-modified LATP particles shows the contribution of SiO_2-y_ (0<y<2) at 102.8 eV. In Fig. S4b, the C *p*_2/3_ spectra of PM-LATP and IB-PM-LATP show contributions at 289, 287, and 284.8 eV that correspond to C=O, C-O, and C-C/C-H, respectively. The spectrum of IB-LATP shows a large peak at 284.8 eV corresponding to C-C/C-H, indicating the successful modification of LATP particles with Si-coupling agents.

**Fig. S4** XPS spectra of (a) Si 2p, (b) C 1s of LATP (black), PM-LATP (red), IB-LATP (blue), and IB-PM-LATP (green).

**Electrochemical impedance spectroscopy of electrolyte/membrane/electrolyte symmetric cells**

For further investigation, the PM-IB-LATP membrane, the LATP plate and TTT-4T were sandwiched between two porous polymer membranes containing EMI and 1M LiTFSI in order to assemble a symmetrical electrolyte/membrane/electrolyte cell. Impedance spectroscopy measurements were performed over the 1 MHz to 100 Hz frequency range. The Nyquist plot for the EMI/composite membrane/EMI in Fig. S5a shows a semicircle and a spike that are attributed to ion transfer in the bulk and grain boundary of the solid electrolyte. The characteristic frequency of the semicircle (20 kHz) is in good agreement with that of the LATP plate. The resistance of the EMI/membrane/EMI cell was 150 Ω cm^-2^, which is slightly larger than that of the Au/membrane/Au cell (115 Ω cm^-2^, Fig. 4) due to the surface resistance between EMI and the membrane. On the other hand, the Nyquist plot for EMI/TTT-4T/EMI (Fig. S5b) shows that TTT-4T exhibits ionically insulating properties, which rules out the possibility of Li-ion transport through the matrix polymer. Therefore, these results confirm Li-ion transport through the IB-PM-LATP particles.

**Fig. S5** Electrochemical impedance spectroscopy data of the EMI (*φ* = 8 mm)/membrane (*φ* = 12 mm)/EMI (*φ* = 8 mm) symmetric electrodes with the frequency from 1 MHz to 100 Hz at 20 °C. (a) Nyquist plot for no membrane (black), the PM-IB-LATP composite membrane (20 μm, red), and LATP plate (260 μm, blue). (b) Nyquist plots for the symmetric cell with TTT-4T (40 μm).

**Battery cell properties**

**Fig. S6** Illustration of Li-air battery cell prepared in this study.

**Table S3.** Weight of the cell components employed in this report

**Battery cell cycle performance**


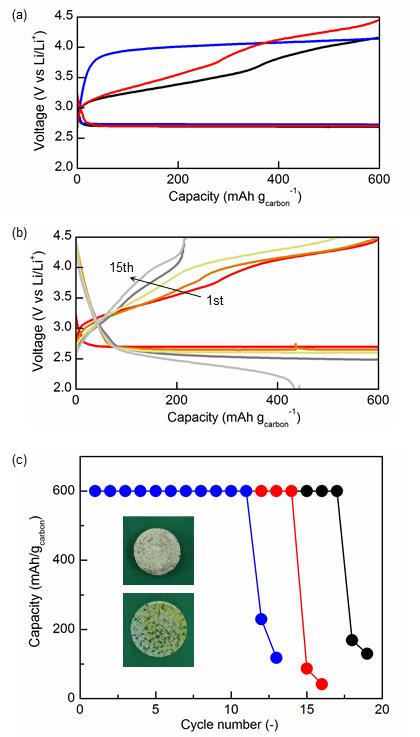


**Fig. S7** Discharge-charge properties of Li-air battery cells with the IB-PM-LATP composite membrane (red), the porous polymer membrane (blue), and the LATP plate (black). (a) Discharge-charge curves of cells with the capacity limit of 600 mAh g^-1^. (b) Selected discharge-charge curves for the composite membrane at the 1st, 5th, 10th, 14th, and 15^th^ cycles. (c) Cycle performance of the cells, and photographs of lithium metal (inset top) and the IB-PM-LATP composite membrane (inset bottom) after cycling.

Because cycle performance of Li-air cell was quite poor when it was fully discharged, the cycle test for the cells with the IB-PM-LATP membrane and the LATP plate was conducted with a capacity limit of 600 mAh g_carbon_^-1^. In addition to the protective separators, the porous polymer membrane used in the electrolyte layer was used as a control sample. The cell condition that gives the maximum specific energy density was applied. The discharge-charge curves of the cells are shown in Fig. S7a. Without the protective separator, the cell with a porous polymer membrane shows a significantly large charge voltage of as high as ~4.0 V. This possibly corresponds to the oxidation of TEGDME that penetrated from the anode to the cathode through membrane pores. The discharge potentials of the cells follow the order: porous polymer membrane > IB-PM-LATP membrane > LATP plate, which corresponds to the conductivities of the membranes. The cell with the IB-PM-LATP membrane retained its discharge-charge capacity over 14 cycles, after which the discharge capacity decreased to < 600 mAh g_carbon_^-1^ at the 15th cycle, whereas the cell with the porous polymer membrane degraded rapidly after the 11th cycle (Fig. S7c). The deterioration phenomenon is similar to that of the LATP plate, which kept the capacity after 17 cycles. The coin cell with the IB-PM-LATP membrane was carefully disassembled and its components observed after cycling (Fig. S7c). The IB-PM-LATP membrane was free of defects and devoid of matrix-polymer swelling. The area resistance of the IB-PM-LATP membrane measured in the symmetrical cell after cycling is 30 Ω cm^2^, which is almost the same as the value before cycling. The surface of lithium metal retained the metallic lustre, implying that the lithium remained electrochemically active. These results suggest that the cycle performance of the Li-air cell is still regulated mainly by the performance of the air cathode.


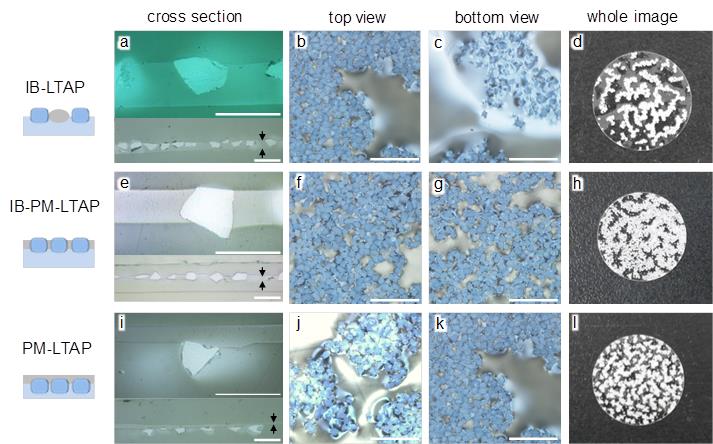
**Optical microscopy images**

**Fig. S8** Optical microscopy images of IB-LATP (a-d), IB-PM-LATP (e-h), and PM-LATP (i-l). Scale bar: (b, c, f, g, j, k) 500 μm, and (a, e, i) 100 μm.

**Peeling test**

LATP plates were cut into rectangles of 5 cm × 1.5 cm, and modified with silane compounds as for the LATP particles. The solution of TTT-4T was coated on the plates, dried, and photo-crosslinked by UV irradiation. Approximately 1 cm of the coated TTT-4T film (thickness: 100 μm) was peeled off, extended using Kapton tape, and gripped by a tensile adhesion machine (LR5KPlus, LLOYD, USA). The peeling strength in the 180° peel test was measured by a tensile machine with a 500-N load cell. The test was run at a peel rate of 5 mm/min for all specimens, and the average peeling strengths were evaluated from the plateau region in Fig. S9. The maximum peeling strength of the interface TTT-4T/PM-LATP is 22 times higher than that of TTT-4T/LATP. This significant increase of adhesion is attributed to the anticipated chemical bonding between the methacrylate group of PM-LATP and the thiol group of TTT-4T. The maximum peeling strength of the interface TTT-4T/IB-PM-LATP is 65% lower than that of TTT-4T/PM-LATP. This decrease in the peeling strength is in accordance with the decrease of methacrylate groups on the surface.

**Fig. S9** Force-displacement curves from the peeling test of the adhesion between the TTT-4T film and LATP plates: LATP (red), PM-LATP (blue), and IB-PM-LATP (black).

**References**

S1. Christensen, J. et al. A critical review of Li/air batteries. *J. Electrochem. Soc.* **159,** R1-R30 (2012)

S2. Goedel, W. A. A simple theory of particle-assisted wetting. *Europhys*. *Lett*. **62**, 607-613 (2003)
